# Quantification of Gait Stability During Incline and Decline Walking: The Responses of Required Coefficient of Friction and Dynamic Postural Index

**DOI:** 10.1155/2022/7716821

**Published:** 2022-10-12

**Authors:** Noor Arifah Azwani Abdul Yamin, Khairul Salleh Basaruddin, Shahriman Abu Bakar, Ahmad Faizal Salleh, Mohd Hanafi Mat Som, Haniza Yazid, Tien-Dat Hoang

**Affiliations:** ^1^Faculty of Electronic Engineering and Technology, Universiti Malaysia Perlis, Pauh Putra 02600, Perlis, Malaysia; ^2^Faculty of Mechanical Engineering and Technology, Universiti Malaysia Perlis, Pauh Putra 02600, Perlis, Malaysia; ^3^Medical Devices and Health Sciences, Sports Engineering Research Center (SERC), Universiti Malaysia Perlis, Pauh Putra 02600, Perlis, Malaysia; ^4^Center of Excellence Automotive & Motorsport (MoTECH), Universiti Malaysia Perlis 02600 Pauh Putra, Perlis, Malaysia; ^5^Faculty of International Training, Thai Nguyen University of Technology, Thai Nguyen, Vietnam

## Abstract

This study aims to investigate the gait stability response during incline and decline walking for various surface inclination angles in terms of the required coefficient of friction (RCOF), postural stability index (PSI), and center of pressure (COP)–center of mass (COM) distance. A customized platform with different surface inclinations (0°, 5°, 7.5°, and 10°) was designed. Twenty-three male volunteers participated by walking on an inclined platform for each inclination. The process was then repeated for declined platform as well. Qualysis motion capture system was used to capture and collect the trajectories motion of ten reflective markers that attached to the subjects before being exported to a visual three-dimensional (3D) software and executed in Matlab to obtain the RCOF, PSI, as well as dynamic PSI (DPSI) and COP-COM distance parameters. According to the result for incline walking, during initial contact, the RCOF was not affected to inclination. However, it was affected during peak ground reaction force (GRF) starting at 7.5° towards 10° for both walking conditions. The most affected PSI was found at anterior-posterior PSI (APSI) even as low as 5° inclination during both incline and decline walking. On the other hand, DPSI was not affected during both walking conditions. Furthermore, COP-COM distance was most affected during decline walking in anterior-posterior direction. The findings of this research indicate that in order to decrease the risk of falling and manage the inclination demand, a suitable walking strategy and improved safety measures should be applied during slope walking, particularly for decline and anterior-posterior orientations. This study also provides additional understanding on the best incline walking technique for secure and practical incline locomotion.

## 1. Introduction

Among the basic movements of the human body is the ability to maintain balance. The vertical projection of the body's COM must be located inside the support area's base in order to ensure stability [1]. Balance control is crucial because a loss of balance might lead to a higher chance of falling [2] and possibly lead to another serious injury. It is difficult on the postural control system to walk uphill. The individual is at a high risk of balance loss and is potential to subsequent falls [3, 4]. In addition, the body is in a continuous imbalance condition during gait [5]. Therefore, maintaining balance is crucial [6] and a challenge in postural control system [1].

There are variety of research that investigate the scope of gait stability. This include age [7, 8], method analysis [9–11], walking strategy [12–16], pathological analysis [17–20], and surface [12, 21–23]. There is also research on stability responses during walking, performed by Amini Aghdam et al. [16] and Blair et al. [24] on camouflaged curb and irregular surface with different walking velocity and speed, respectively. While, Schut et al., examine dynamic stability and dynamic loading on outdoor surface during gait [22].

To date, the studies on postural balance control and stability during walking on incline plane that is accessible to the public provide limited amount of information. In addition, only a few studies had explored dynamic stability of slope gait by assessing both kinematic and kinetic parameters. For instance, Vierra et al. explore gait postural stability on incline surface using a method that only involves kinematic parameter instead of both kinetic and kinematic parameters [25]. In addition, studies on incline plane are not only limited, but the walking dynamic stability is assessed via the use of a treadmill. According to Park et al., incline locomotion study using a treadmill might come out with slightly different mechanics of the movement compared to ground locomotion [26]. On the other hand, a customized incline platform should be used instead for a better over ground imitation. Moreover, most of previous studies on incline gait determine the center of mass (COM) to explore the dynamic stability using full body marker set [27, 28] which is costly and time consuming.

Currently, studies on dynamic stability of slope gait by assessing kinematic and kinetic parameters is limited. What remains unknown is that the angle of slope surface will affect stability during incline and decline walking. This study is constructed to gain further understanding on the influence of incline and decline walking towards gait stability in terms of required coefficient of friction (RCOF), postural stability index (PSI), and center of pressure (COP)-center of mass (COM) distance under specific angles of surface inclination. This study implements the sacral marker method which reduces time consuming and simplifies the analysis process in determining the COM position for COP-COM distance measurement where no previous information on this method applied on incline walking was previously provided.

## 2. Methods

### 2.1. Participants

Twenty-three volunteers with a normal body mass index (BMI) and an age of 24 ± 1.2 years took part in this study. In order to avoid varying differences in mobility, participants that struggle to perform during walking activity or experience musculoskeletal injury or orthopedic abnormalities were avoided from this research. Signed consent was collected before the experiments were conducted. Ethic Committee of the Universiti Malaysia Perlis (UniMAP) approved the experiment in this study.

### 2.2. Equipment and Devices

**Figure 1:**
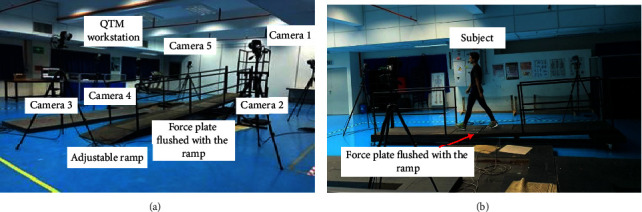
(a) Equipment layout of the experiment (b) Locomotion of the subject on the ramp.

As illustrated in Figure 1, the ramp used in this study is a custom-built wooden platform with adjustable angle. The ramp was customized to allow the two force plates to be completely leveled with the walking surface. The dimension of the slope is 5 m long and 1 m wide. There are two spaces incorporated into the slope that will allow for the insertion of force plates on the walking surface. The inclination of the platform can be altered to 0°, 5°, 7.5°, and 10°.

Five motion capture cameras (Qualysis, Gothenburg) and two force plates (Bertec Corp., Ohio) were used in this study. The experimental layout and walking demonstration on the ramp are shown in Figure 1.

**Figure 2:**
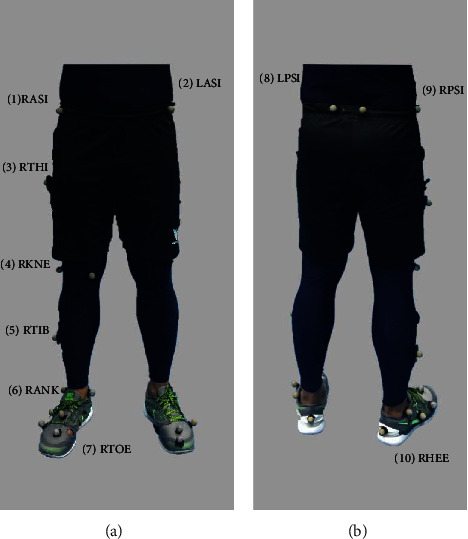
Marker's position on the subject's body in (a) front view; (b) rear view.

The lower limb “Plug-in Gait” marker set was modified and used in this investigation to put ten reflecting markers (20 mm in diameter). The subjects' right limb was where the markers were positioned.  Figure 2(a) shows the marker placements on the anterior part of the subject's body while Figure 2(b) shows the marker placements on the posterior part of the subject's body.

Motion capture equipment (Qualysis, Gothenburg, Sweden) was used to capture the motion trajectories of the markers. After the system managed to capture this motion, it was sent to a visual three-dimensional (Visual3D) software (C-motion, Germantown) before transferring them to a Matlab (2019) software to procure the RCOF and PSI. The shoes were modified so that the markers could be seen and can be attached directly to the foot instead of the shoe.

### 2.3. Procedure

Before beginning the experiment, the anthropometry information of the participants, including their height, weight, and widths of the knee and ankle, was gathered. The neutral position of the joint was obtained using a reference static trial, where the subjects stood straight in a double-leg support posture [24]. Following the static data collection, the subjects then were asked to walk across/along the ramp (uphill and downhill) that is set for different angles (0°, 5°, 7.5°, and 10°), with their preferred comfortable speed wearing the same types of shoes. The trial was deemed successful if all markers were easily seen and foot-to-force plate contact was made without discernible changes in gait.

### 2.4. Data Analysis

Using the Qualysis Track Motion system, kinematic and force plate data were gathered and analysed. The GRF was acquired after the data were imported and converted using the Visual3D. In this experiment, the musculoskeletal model used was a standard model obtained from the plug-in lower limb. “Plug-in Gait” then, calculates the location and orientation of each segment using a direct (nonoptimal) pose estimation method. The RCOF, PSI, and COP-COM distance parameters were then calculated using a custom-written Matlab software (R2019a; Mathworks, USA). The trajectories of reflecting markers were low-pass filtered at 6 Hz and the obtained data were examined for total stance phase [23]. The GRF pattern was used to determine when the stance phase began and ended.

#### 2.4.1. Required Coefficient Friction (RCOF)

RCOF is the minimal coefficient of friction required to prevent slip initiation at the shoe-floor contact. The RCOF was calculated by dividing the vertical GRF with the resultant of horizontal GRF. The RCOF can be expressed in mathematical form as (1).

(1) $$\begin{array}{c} RCOF=\;\frac{\surd \left(F_{AP}^{2}+F_{ML}^{2}\right)}{F_{V}}. \end{array}$$

*F *_*AP*_ = GRF in anterior-posterior.

*F *_*ML*_ = GRF in medial-lateral.

*F *_*V*_ = Vertical GRF.

#### 2.4.2. Dynamic Postural Stability Index (DPSI)

A combination of anterior-posterior (AP), medial-lateral (ML), and vertical GRFs make up the DPSI. Moreover, it will also provide stability indices for all directions. While the MLSI and APSI evaluate variations along the *x*- and *y*-axes of the force plate, respectively. The VSI measures the variation from the subject's body weight, which is comparable to the typical vertical GRF along the force plate's *z*-axis. The APSI, MLSI, VSI, and DPSI were obtained using formula as shown in Table 1 [29].

#### 2.4.3. Distance of Center of Pressure (COP) and Center of Mass (COM)

The combination of kinetic of COP and kinematic COM trajectories in terms of COP-COM distance had been used as the state of balance indicator. The COP was determined by visual 3D software and the COM was collected based on sacrum position for each subject [30]. The COP and COM in AP and ML directions were identified by calculating the difference between maximum and minimum amplitude which can be formulated as shown in (2) and (3), respectively:

(2) $$\begin{array}{c} COP=\;\frac{\text{max}\left(COP\right)-\text{min}\left(COP\right)}{l_{0}}, \end{array}$$

(3) $$\begin{array}{c} COM=\;\frac{\text{max}\left(COM\right)-\text{min}\left(COM\right)}{l_{0}}, \end{array}$$

maxCOP = the maximum displacement of mean COP.

minCOP = the minimum displacement of mean COP.

maxCOM = the maximum displacement of mean COM.

minCOM = the minimum displacement of mean COM.

*l*_0_ = original leg length

The peak-to-peak COP-COM distance of the COP and COM displacement in AP and ML directions was calculated using (4) and (5), respectively.

(4) $$\begin{array}{c} COP-COM\;distance_{AP}=RMS\left|COP-COM_{AP}\right|, \end{array}$$

(5) $$\begin{array}{c} COP-COM\;distance_{ML}=RMS\left|COP-COM_{ML}\right|. \end{array}$$

*COM *_*AP*_ = center of mass in anterior-posterior.

*COM *_*ML*_ = center of mass in medial-lateral.

### 2.5. Statistical Analysis

The Shapiro–Wilk normality test was employed to obtain the means and standard deviations of the gathered parameters. The parametric and nonparametric test were utilized for normal and abnormal data, respectively. The statistical analysis was carried out to compare the situations of level and incline/decline walking using one-way ANOVA (parametric test) and Kruskall–Wallis (nonparametric test), with statistical significance set at *p* < 0.05. Both tests were selected to look at the differences between two or more means for both normally distributed and abnormally distributed data. The statistical study was completed using IBM's Statistical Package for Social Science (SPSS).

## 3. Results

### 3.1. Required Coefficient of Friction (RCOF)

The first aim of this study is to explore the effect on required coefficient of friction (RCOF) during incline/decline walking. The RCOF investigated was observed during initial foot contact or heel strike of gait cycle and during maximum or peak of GRF produced.  Table 2 shows the RCOF is not sensitive to the inclination during initial foot contact for incline walking. Instead, during decline walking on the other hand, the RCOF altered, starting from 7.5° towards 10° (*p* < 0.05). However, the RCOF obtained during peak GRF is affected at 7.5° towards 10° inclination during incline walking but it was not affected to inclination during decline walking where no significant differences were found (*p* > 0.05) which is in contrast to RCOF during initial foot contact.

### 3.2. Postural Stability Index

Another parameter explored in this study is postural stability.  Table 3 presents the postural stability index responses to the surface inclination. With respect to the anatomical coordinate system, the postural stability index investigated in the present study is the stability index in anterior-posterior, medial-lateral, and vertical directions. The dynamic stability index (DPSI) was also investigated, which is the composite of the APSI, MLSI, and VSI.

According to Table 4, during inclined walking, the APSI was affected at 5° towards 10° inclination where there is statistically significant difference compared to level (0°) walking (*p* < 0.05). However, MLSI was only affected at 10° of inclined walking. Instead, the VSI and DPSI were not affected by surface inclination during incline walking. No significant difference was obtained (*p* > 0.05) for all inclination angles.

As demonstrated in Table 4, the APSI was affected to some extent by surface slope during decline walking, similar to incline walking, as there is a significant difference in comparison to 0° inclination (*p* < 0.05). Furthermore, both the MLSI and VSI are affected at 7.5° towards 10° inclination during decline walking. However, the DPSI was not affected to the surface inclination for all slope angles (*p* > 0.05) during decline walking.

### 3.3. COP-COM Distance

Table 5 lists the results of RMS of COP-COM distance throughout the stance phase of gait cycle in anterior-posterior and medial-lateral directions. The COP-COM_AP_ distance was affected by surface inclination at 7.5° towards 10° inclination during incline walking as presented in Table 5. In addition, the COP-COM_AP_ distance seemed to be influenced by surface inclination at 5° inclination during declined walking as there is significant difference found compared to level walking (*p* < 0.05). On the other hand, for both incline and downhill walking, the COP-COM_ML_ distance was only impacted by surface inclination at 10° (*p* < 0.05).

## 4. Discussion

The stability response towards surface inclination during incline/decline walking is the main purpose of this study. The gait stability was explored with reference to kinetic and kinematic parameters. GRF as kinetic parameters was expanded into three directions (AP, ML, and vertical) which was utilized to investigate the stability in terms of RCOF and PSI. In addition, the stability was studied as well, by combining the kinetic (COP) and kinematic (COM) parameter to obtain COP-COM distance.

One interesting finding was that, during incline walking, the RCOF was affected at 7.5° towards 10° when the GRF reached the maximum value. These results support the idea of Yamaguchi et al., where the RCOF is affected at the push-off phase of gait cycle during level walking [31]. On the other hand, during decline walking, the RCOF was affected to surface inclination at 7.5° towards 10° inclination when the foot initial touches. A possible explanation of these findings is, the common RCOF has two peaks and one valley. The peaks denote the phases in which shear forces are greater; the first occurs during the first contact phase, and the second occurs during the push-off phase [32]. In addition, the observed effect of the RCOF might be explained by the fact that, in gait cycle, peak GRF can be observed during loading and push-off phase [33]. Friction was affected during this phase by the anterior-posterior force since there is a minimum (or maximum) braking peak [34]. Slips can happen when the footwear-floor interface's frictional qualities are insufficient to counteract the biomechanical demands of walking [35].

Even at the lowest inclination angle of 5°, the stability in terms of PSI was found to be the most susceptible to the surface inclination in anterior-posterior direction (APSI) during both incline and decline walking. These findings are in line with recent research that revealed that during both planned and unexpected gait termination, those with chronic ankle instability had higher APSI scores than controls [36].

Similarly, based on the result of COP-COM distance evaluated, it is found that COP-COM distance is more affected in anterior-posterior direction as well. The result showed that, COP-COM distance is affected by the incline surface, starting at 7.5° and even 5° inclination in anterior-posterior direction during incline and decline walking, respectively, instead of at 10° inclination only for both walking condition in medial-lateral direction. This result corroborates the findings of Viera et al., who demonstrated that gait beginning on inclined surfaces showed significant variations in the AP direction for COP excursion but not in the ML direction [37]. A possible explanation for this might be that, the body requires to counteract the gravitational effect that pushes and pulls the body in the opposite and similar direction during the incline and decline walking, respectively. These gravitational effects are related to the forward and backward fall which was during anterior-posterior direction [38]. It is also consistent with the findings by Ma et al., where they found that the walking height and width could influence the gait parameters [39]. Furthermore, forward momentum might've increased because of gravity. The stability or balance control is affected as the forward momentum increases, which weakens the control of anterior-posterior movement [40].

Another notable finding is that, based on statistically significant differences (*p* < 0.05), the stability of postural index was shown to be more impaired during decline walking rather than incline walking of APSI, MLSI, and VSI. During decline walking, as mentioned earlier, APSI was found to be sensitive towards all surface inclination (5°.7.5° and 10°). While both, the MLSI and VSI were found to be affected at 7.5° until 10° inclination. In addition, the COP-COM distance was affected during decline walking since there is significant difference (*p* < 0.05) observed from 5° towards 10° inclination in comparison to incline walking where significant difference was found starting from 7.5° towards 10° inclination. In accordance with this present results, previous study showed that, for both incline and decline walking, there are changes in COM and COP with the latter demonstrating larger changes of the task [37, 41].

The main cause for these findings is that the body is exposed to an increasing downward gravitational shear force during downhill walking with increasing inclination angle. Hence, there will be increase in demand at the lower limb joints, which is attributed by this shear force which must be met by forces and moments generated by the relevant muscles in order to maintain proper posture and movement [42]. A well-controlled coordination of joint involved in encountering the different surface inclination with various kinematic configurations is required. Moreover, the alteration of foot in gait strategy during decline walking is found to differ from incline walking [43]. Thus, the PSI and COP-COM distance is related to the force generated, along with kinematic configuration. These factors may explain the different response of PSI and COP-COM distance during incline and decline walking.

In addition, during decline walking, a more conservative gait style as strategy control was found [44] which could be used to reduce the danger of falling while there is an increase in energy consumption [45, 46]. However, the gait strategy applied during incline walking will reduce the energy expenditure as metabolic demand is high during incline walking.

There are several limitations that was acknowledged in this study. First, the best marker position of each participant selected might not be similar as there are three trials taken for every incline angle condition. However, it will not affect the overall result because the measurement applied for all 23 participants and the average value was collected. Second, all participants were asked to walk at their comfortable walking speed during the experiment. Therefore, the speed might be different among participants. However, it is ensured the speed was within the specified range so there is no obvious difference observed.

## 5. Conclusion

This study was set out to gain a better understanding of the effect of surface inclination on stability during slope walking for both directions; incline and decline. The finding of this study has shown that, the stability in terms of RCOF was affected by the surface inclination at initial foot contact, while peak GRF was affected during decline walking and incline walking, respectively. The second major finding was the stability parameters. In terms of PSI and COP-COM distance, the stability parameters revealed to be more affected by surface slope angle for decline walking rather than incline walking in anterior-posterior direction, especially based on statistically significant differences obtained in APSI and COP-COM_AP_ parameters. The result of this study provided insight on the changes of stability parameter due to gait strategy alteration in order to provide a safe environment and walking style on slope. In addition, from the result obtained, a better safety measure was obtained with suitable walking strategy to prevent from falling and coping with the inclination demand due to gravitational force and friction, especially during decline walking. The practical application that might be related to the findings obtained from this study, for example, is the industrial area where the workers are exposed to the incline surface, especially in manufacturing and building construction sector. The results found are beneficial to prevent accident or injury during works. In summary, the result of this study has provided additional understanding on the proper walking strategy on a slope surface for a safer and comfortable incline movement. The surface inclination affects stability in terms of RCOF, PSI, and COP-COM distance. This study also focused on the stability of individuals with normal BMI during their walks on slope surface. The effect of surface inclination on people with a body mass index (BMI) over 25 should be further studied.

## Figures and Tables

**Table 1:** Calculation formulas for the APSI, MLSI, VSI, and DPSI.

Variable	Equation	
APSI	$=\;\sqrt{\sum^{}{\left(0-GRFx\right)}^{2}/num\;of\;data\;points÷body\;weight}$	(2)
MLSI	$=\;\sqrt{\sum^{}{\left(0-GRFy\right)}^{2}/num\;of\;data\;points÷body\;weight}$	(3)
VSI	$=\;\sqrt{\sum^{}{\left(Body\;weight-GRFz\right)}^{2}/num\;of\;data\;points÷body\;weight}$	(4)
DPSI	$=\;\sqrt{\sum^{}{\left(0-GRFx\right)}^{2}+\;\sum^{}{\left(0-GRFy\right)}^{2}+{\left(Body\;weight-GRFz\right)}^{2}/num\;of\;data\;points÷body\;weight}$	(5)

**Table 2:** RCOF during initial foot contact and peak GRF for incline and decline walking.

Phase of gait cycle	Degree of inclination (°)	Incline walking	Decline walking
		RCOF (mean ± SD)	RCOF (mean ± SD)
Initial foot contact	0.0	0.192 ± 0.103	0.192 ± 0.103
	5.0	0.156 ± 0.104	0.247 ± 0.178
	7.5	0.689 ± 1.172	**0.645** **±** **0.298**^*∗*^
	10.0	0.300 ± 0.175	**0.456** **±** **0.152**^*∗*^
Peak GRF	0.0	0.163 ± 0.029	0.163 ± 0.029
	5.0	0.214 ± 0.079	0.129 ± 0.093
	7.5	**0.246** **±** **0.038**^*∗*^	0.144 ± 0.088
	10.0	**0.307** **±** **0.024**^*∗*^	0.116 ± 0.071

^*∗*^represent significantly different in comparison to level walking with *p*-value <0.05.

**Table 3:** Postural stability index during incline walking.

Degree of inclination (°)	APSI	MLSI	VSI	DPSI
	(Mean ± SD)	(Mean ± SD)	(Mean ± SD)	(Mean ± SD)
0.0	0.822 ± 0.183	0.608 ± 0.078	7.267 ± 0.623	4.725 ± 0.738
5.0	1.217 ± 0.152^*∗*^	0.567 ± 0.214	7.211 ± 0.632	4.347 ± 0.449
7.5	1.238 ± 0.152^*∗*^	0.510 ± 0.146	7.165 ± 0.293	4.200 ± 0.531
10.0	1.817 ± 0.202^*∗*^	1.209 ± 0.370^*∗*^	6.670 ± 0.657	4.096 ± 0.427

^*∗*^represent significantly different in comparison to level walking with *p*-value <0.05.

**Table 4:** Postural stability index during decline walking.

Degree of inclination (°)	APSI	MLSI	VSI	DPSI
	(Mean ± SD)	(Mean ± SD)	(Mean ± SD)	(Mean ± SD)
0.0	0.822 ± 0.183	0.608 ± 0.078	7.267 ± 0.623	4.725 ± 0.738
5.0	1.710 ± 0.449^*∗*^	0.302 ± 0.156	6.798 ± 0.527	4.501 ± 0.661
7.5	1.259 ± 0.554^*∗*^	1.244 ± 0.389^*∗*^	5.071 ± 1.022^*∗*^	4.401 ± 0.612
10.0	1.931 ± 0.657^*∗*^	0.275 ± 0.053^*∗*^	6.025 ± 0.188^*∗*^	4.475 ± 0.802

**Table 5:** The RMS of COP-COM distance in AP and ML.

Degree of inclination (°)	COP-COM_AP_ (mean ± SD)		COP-COM_ML_ (mean ± SD)	
	Incline walking	Decline walking	Incline walking	Decline walking
0.0	0.192 ± 0.009	0.192 ± 0.009	0.089 ± 0.020	0.089 ± 0.020
5.0	0.204 ± 0.052	0.273 ± 0.064^*∗*^	0.104 ± 0.015	0.073 ± 0.023
7.5	0.158 ± 0.023^*∗*^	0.224 ± 0.030^*∗*^	0.074 ± 0.013	0.073 ± 0.023
10.0	0.183 ± 0.014^*∗*^	0.325 ± 0.022^*∗*^	0.121 ± 0.025^*∗*^	0.067 ± 0.009^*∗*^

^*∗*^represent significantly different in comparison to level walking with *p*-value <0.05.

## Data Availability

The data used to support the findings of this study are available from the corresponding author upon request.
